# One Size Fits All: Does the Dogma Stand in Radiation Oncology?

**DOI:** 10.1016/j.ebiom.2016.07.025

**Published:** 2016-07-20

**Authors:** David Azria, Celine Bourgier, Muriel Brengues

**Affiliations:** a Department of Radiation Oncology, Montpellier Cancer Institute (ICM), Montpellier Cancer Research Institute (IRCM), University of Montpellier, Montpellier, France; b INSERM U1194, Montpellier Cancer Research Institute (IRCM), University of Montpellier, Montpellier, France

External beam radiotherapy (EBRT) is the most treatment used in solid tumors as nearly 50% of cancer patients receive curative EBRT in the world. Its success depends mainly on the total dose homogeneously delivered within the target volume. Nevertheless, EBRT inevitably exposes surrounding normal tissues and may cause late and sometimes irreversible toxicities depending on different cells or tissues (stroma, vascular, parenchymal, immune cells). Interactions between cells or compartmental tissues and the immune system via cytokines produce inflammatory and pro-fibrotic reactions. Cell depletion, inflammation, repopulation and remodeling are reminiscent of the wound healing process leading to different severities of late deterministic effects (Herskind et al., 2016).

Stratifying patients according to the toxicity risk and modulating EBRT dose would provide a valuable tool for personalized EBRT (Barnett et al., 2009, Bourgier et al., 2015). Many efforts have been made to develop assays capable of predicting susceptibility for the development of radiation injury that finally allow customization of EBRT protocols on an individual basis.

Indeed and as presented in this current volume of *EBioMedicine*, Kerns and colleagues (Kerns et al., 2016), aimed to meta-analyze individual level data from four genome-wide association studies from prostate cancer radiotherapy cohorts including 1564 men to identify novel genetic markers of toxicity. A fixed-effects meta-analysis identified two SNPs: rs17599026 on 5q31.2 with urinary frequency and rs7720298 on 5p15.2 with decreased urine stream. These SNPs lie within genes that are expressed in tissues adversely affected by pelvic radiotherapy including bladder, kidney, rectum and small intestine. The authors mentioned that new moderate-penetrance genetic variants associated with radiotherapy toxicity have been identified. As we know, radiogenomics (RG) attempts to link germ line genotypic variations and clinical variability observed after EBRT. The aim of RG is to identify the alleles that underlie the inherited dissimilarities in phenotype (Rosenstein et al., 2014). However, this hypothesis does not assume that all of the phenotypic differences are due to germ line genetic alterations, but also epigenetic changes and other factors such as systemic treatment or tobacco use. Recently, DNA methylation profiling of dermal fibroblasts obtained from breast cancer patients prior to irradiation identified differences associated with fibrosis. One region was characterized as a differentially methylated enhancer of diacylglycerol kinase alpha (DGKA). Decreased DNA methylation at this enhancer was shown to enable recruitment of the profibrotic transcription factor early growth response 1 (EGR1) and then capable to facilitate radiation-induced DGKA transcription in cells from patients later developing fibrosis. Conversely, inhibition of DGKA showed pronounced effects on diacylglycerol-mediated lipid homeostasis with profibrotic fibroblast activation (Weigel et al., 2016).

As mentioned in a recent review by Herskind et al. (2016), pathway analyses incorporating different ‘omics’ approaches may be more efficient in identifying critical pathways than those based on single ‘omics’ data sets. Integrating these pathways with functional assays may be powerful in identifying multiple subgroups of EBRT patients characterized by different mechanisms. In that way, monocentric cohorts suggested that radiation-induced CD8 T-lymphocyte apoptosis (RILA) as a functional test can predict late toxicity after curative intent EBRT. We recently assessed the role of RILA as a predictor of breast fibrosis (bf +) after adjuvant breast EBRT in a prospective multicenter trial (Azria et al., 2015). A total of 502 breast-cancer patients (pts) treated by conservative surgery and adjuvant EBRT were recruited at ten centers. RILA was assessed before EBRT by flow cytometry. Impact of RILA on bf + (primary endpoint) or relapse was assessed using a competing risk method. With a median follow-up of 38.6 months, grade ≥ 2 bf + was observed in 64 pts (14%). A decreased incidence of grade ≥ 2 bf + was observed for increasing values of RILA (p = 0.012). No grade 3 bf + was observed for patients with RILA ≥ 12%. Negative predictive value for grade ≥ 2 bf + was equal to 91% for RILA ≥ 20% where the overall prevalence of grade ≥ 2 bf + was estimated at 14%. A significant decrease in the risk of grade ≥ 2 bf + was found if patients had no adjuvant hormonotherapy (sHR = 0.31, p = 0.007) and presented a RILA ≥ 12% (sHR = 0.45, p = 0.002). Different hypotheses to understand the mechanisms of inverse correlation between low radiation response of lymphocytes and the increase risk of developing late reaction after EBRT are currently under investigations: (i) Production of cytokines and inflammatory immune cells attraction to the irradiated tissue (Azria et al., 2008); (ii) Protein and ROS production modification, enhanced genomic instability, terminal differentiation of fibroblasts and increased risk of fibrogenesis (Lacombe et al., 2013); (iii) genetic defect in the DNA damage response, DNA repair reduction, increased genomic instability and increased premature terminal differentiation of fibroblasts (Herskind et al., 2016).

Clinical implementations with interventional protocols are starting using this assay permitting distinction between patients without any over-risk of toxicity (considered as resistant to late effects) and patients clearly at risk of developing more late effects defined as very sensitive (Barnett et al., 2015). In terms of altered management, hyperfractionation can reduce toxicity with no risk of loss of local control or to allow for dose escalation in very sensitive patients. For more resistant patients, an increase in dose should be possible and hypofractionation regimen should be largely proposed leading to a medicoeconomical improvement of our treatments. This might be in favor of adding novel targeted or existing systemic therapies (Barnett et al., 2015).

In conclusion, there is no doubt that personalized radiotherapy driven by companion tests of radiotoxicity but also of tumor radioresponse will be the standard of care in the near future as it is already the case for targeted therapies in medical oncology. One size will no longer fit all!

## Disclosure

The authors declare that they have no competing interest regarding this manuscript.
